# The Angiotensin-Converting Enzyme Inhibitor Lisinopril Mitigates Memory and Motor Deficits in a *Drosophila* Model of Alzheimer’s Disease

**DOI:** 10.3390/pathophysiology28020020

**Published:** 2021-06-18

**Authors:** Jimiece Thomas, Haddon Smith, C. Aaron Smith, Lori Coward, Gregory Gorman, Maria De Luca, Patricia Jumbo-Lucioni

**Affiliations:** 1 McWhorter School of Pharmacy, Samford University, Birmingham, AL 35229, USA; jthoma17@samford.edu (J.T.); hsmith16@samford.edu (H.S.); csmith67@samford.edu (C.A.S.); 2 Pharmaceutical Sciences Research Institute, McWhorter School of Pharmacy, Samford University, Birmingham, AL 35229, USA; lcoward@samford.edu (L.C.); ggorman@samford.edu (G.G.); 3 Pharmaceutical, Social, and Administrative Sciences, McWhorter School of Pharmacy, Samford University, Birmingham, AL 35229, USA; 4 Department of Nutrition Sciences, School of Health Professions, University of Alabama, Birmingham, AL 35233, USA; mdeluca2@uab.edu; 5 Department of Biology, College of Arts and Sciences, University of Alabama, Birmingham, AL 35233, USA

**Keywords:** angiotensin-converting enzyme inhibitors, Alzheimer’s disease, Drosophila, aging, kynurenine pathway of tryptophan metabolism

## Abstract

The use of angiotensin-converting enzyme inhibitors (ACEis) has been reported to reduce symptoms of cognitive decline in patients with Alzheimer’s disease (AD). Yet, the protective role of ACEis against AD symptoms is still controversial. Here, we aimed at determining whether oral treatment with the ACEi lisinopril has beneficial effects on cognitive and physical functions in a *Drosophila melanogaster* model of AD that overexpresses the human amyloid precursor protein and the human β-site APP-cleaving enzyme in neurons. We found a significant impairment in learning and memory as well as in climbing ability in young AD flies compared to control flies. After evaluation of the kynurenine pathway of tryptophan metabolism, we also found that AD flies displayed a >30-fold increase in the levels of the neurotoxic 3-hydroxykynurenine (3-HK) in their heads. Furthermore, compared to control flies, AD flies had significantly higher levels of the reactive oxygen species (ROS) hydrogen peroxide in their muscle-enriched thoraces. Lisinopril significantly improved deficits in learning and memory and climbing ability in AD flies. The positive impact of lisinopril on physical function might be, in part, explained by a significant reduction in ROS levels in the thoraces of the lisinopril-fed AD flies. However, lisinopril did not affect the levels of 3-HK. In conclusion, our findings provide novel and relevant insights into the therapeutic potential of ACEis in a preclinical AD model.

## 1. Introduction

Human average life expectancy continues to grow in many industrialized countries [1]. An inevitable consequence of the rise in life expectancy is the increase in the number of people affected by dementia, a syndrome that is mainly characterized by progressive deterioration of cognitive functions, such as memory loss [2]. Alzheimer’s disease (AD) is the most common age-related form of dementia, accounting for approximately 60–80% of dementia cases, and the sixth leading cause of death among senior adults in the US [3]. An estimated 5.8 million seniors in the US are living with AD [3] and this number is projected to nearly triple by 2050 [4]. Yet, efforts to identify effective therapies for the prevention and treatment of AD have been unsuccessful [5].

In recent years, it has become clear that pharmacological inhibition of the renin–angiotensin system (RAS) can favorably impact the age-associated functional decline of various tissues and organs, including the brain [6,7,8]. This is due to the presence of several RAS components in almost every organ (local RAS), where they exert diverse organ-specific physiological and pathophysiological functions [9,10]. Local RASs operate independently of each other and of the circulating RAS whose primary function is to regulate arterial pressure as well as water and sodium homeostasis through the action of angiotensin effector peptides, such as angiotensin (Ang) II, Ang III, and Ang 1–7 [11]. The main effector of both circulating and local RAS is Ang II that is produced from Ang I by the action of the angiotensin-converting enzyme (ACE). Ang II exerts its actions by binding with equal affinity to two main receptors, type 1 (AT_1_) and type 2 (AT_2_), which induce several opposite intracellular events [11].

A growing body of epidemiological and experimental evidence suggests a potential role of the brain RAS in the development and progression of AD, with its inhibition, through ACE inhibitors (ACEis) or AT_1_ receptor blockers (ARBs), reducing AD signs and symptoms [12]. For example, findings from a randomized, prospective, parallel-group trial revealed that administration of the ACEi captopril or perindopril could slow the rate of cognitive decline in mild to moderate AD patients in comparison with other antihypertensive drugs [13]. Moreover, hypertensive participants who were users of ARBs or ACEis showed better preservation of memory over time than those taking other antihypertensive medications in a recent cross-sectional study [14]. The beneficial effect of ACEis on AD has also been observed in animal models of AD, with the neuroprotective profile induced by the drug being accompanied by reduced amyloidogenic processing of the amyloid precursor protein (APP) [15]. However, the latter observation has been contradicted by studies reporting that ACE reduces amyloid β (Aβ) deposition [16]. Thus, the protective role of ACEis against AD symptoms is still controversial and further work is needed in this area.

In this study, we used *D. melanogaster* to gain knowledge into the relationship between ACEi therapy and AD. *D. melanogaster* is an attractive model for several reasons. First, *Drosophila* has long been recognized as a useful model to study the fundamental processes underlying health and disease, including neurodegenerative diseases [17]. For instance, using a *Drosophila* AD model, Coelho et al. [18] were the first to demonstrate that removal of less fit neurons through apoptosis is beneficial since it delays β-amyloid-induced brain damage. Second, given that RAS blockers are by design blood pressure medications that impact blood flow to organs/tissues, disentangling the vascular hemodynamic effects of the ACEi drugs from their direct effects on cellular processes remains a challenge in humans and in vivo vertebrate models. The use of an invertebrate model with an open circulatory system, such as *D. melanogaster* is, therefore, likely to provide essential insights into the mechanisms through which RAS blockade drugs function. Third, components of the RAS system are evolutionarily conserved, with the critical parts of the system first appearing in primitive chordates and tunicates [19]. *Drosophila* orthologs of human ACE, called AnCE and angiotensin-converting enzyme-related (ACER), respectively, have been described [20], and the activity of AnCE is inhibited by the same drugs that inhibit human ACE, such as captopril and lisinopril, through a similar mechanism [21]. Fourth, recent data revealed that long-term treatment with lisinopril preserves physical resilience with aging and extends lifespan in *D. melanogaster* [22]. Finally, captopril has been reported to rescue memory defects in a *Drosophila* model of AD, with no effects on the amyloid pathway [23].

It is well-recognized that slow motor performance is associated with cognitive impairment in elderly people, including AD patients [24]. However, it remains unclear whether the same pharmacological therapy might have beneficial effects on both cognition decline and motor dysfunction. To this end, the primary objective of this study was to assess the potential effects of the ACEi lisinopril on the decline in memory and climbing ability occurring in a *Drosophila* model of AD.

## 2. Materials and Methods

### 2.1. Drosophila Genetics

A *Drosophila* line (Bloomington Stock Center# 33798) overexpressing the human amyloid precursor protein (*hAPP*) and the β-site APP-cleaving enzyme (*hBACE*) was used as an AD model (i.e., *hAPP*, *hBACE*/*+*; *elav-gal4*/*+*). Phenotypic findings in this model resemble those observed in mammalian AD models with marked locomotion impairments and increased oxidative stress [25]. A tissue-specific driver, *elav-gal4*, was used to target transgene expression neuronally. Bloomington stock *w^1118^* (# 5905) was used to generate driver-alone controls (i.e., *elav-gal4*/*+*). All experimental flies were kept in vials containing 10 mL of standard cornmeal, agar, molasses, and yeast medium, at a constant temperature of 30 °C, 60–75% relative humidity, and 12/12 h light/dark cycle. Lisinopril dihydrate (AvaChem Scientific, San Antonio, TX, USA) was dissolved in water then added to molasses-based food to perform the experiments described below. Male virgin flies were either fed a standard medium or received 1 mM lisinopril shortly after eclosion and for the entire duration of the experiment. We chose 1 mM as the intervention dose based on prior literature [22,26] showing that this dose had a positive impact on postponing the age-associated decline of various traits in wild-type *Drosophila* cohorts. Flies were transferred to new food every 4–5 days.

### 2.2. Measurement of Lisinopril Concentration

Liquid chromatography–tandem mass spectrometry (LC–MS/MS) was used to confirm drug uptake in our control and transgenic fly cohorts (see Supplementary Figure S1). Tissues were homogenized in 5 mM ammonium acetate buffer (Fisher Scientific, Waltham MA, USA). An aliquot of 50 μL was used for analysis. Calibration standards, blanks, and quality controls (QCs) were prepared by spiking naïve homogenate (100 µL) with the appropriate amount of lisinopril with concentrations in the tissue homogenate ranging from 5 to 20,000 ng/mL. Samples and standards were fortified with an internal standard,5 µL of 100 ng/mL Enalaprat (Cayman Chemical, Ann Arbor, MI, USA) in DI water, and extracted with 0.5 mL 90:10 methanol: acetone (Fisher Scientific, Waltham, MA, USA). Samples were vortexed well, centrifuged and the supernatant was evaporated under nitrogen at 50 °C. Samples were redissolved in 200 µL DI water and transferred to limited-volume autosampler vials for analysis by LC–MS/MS. Detection was accomplished using an Applied BioSystems 4000 QTRAP (Applied Biosystems, Foster City, CA, USA) triple quadrupole mass spectrometer equipped with an electrospray ionization source operated at a potential of 5 kV at 450 °C operating in the MRM mode. Data were collected using Analyst 1.6.2 (Applied Biosystems, Foster City, CA, USA) and normalized to protein concentration.

### 2.3. Learning and Memory Assay

Control and transgenic male flies either treated with a 1 mM lisinopril or untreated underwent an aversive phototaxic suppression assay as described previously [27]. This assay has been described as a simple and effective way to characterize memory deficits in a fly model of neurodegenerative disorder [28]. Flies, normally attracted to light, were trained to change preference to dark by pairing a light stimulus with an aversive odor, 4-methylcyclohexanol (MCH; Tokyo Chemical Industry, Portland, OR, USA). After 5–7 days of lisinopril treatment, cohorts of ~30 treated and untreated flies (i.e., 5–7 days old) were quickly transferred to an opaque training chamber with an open and perforated section to control light and odor exposure under controlled conditions of temperature and humidity. Replicates of the same cohort were run at the same time of the day. Olfactory deficits were not detected in control and AD flies (data not shown). Each fly cohort was allowed two minutes for acclimation before beginning exposure to one minute of high-intensity light paired with MCH followed by 10 min of darkness with no odor. This training regimen was repeated for 10 cycles and conducted under red lighting as the wavelength is beyond the flies’ spectrum of vision. An air pump was used to push either the odor or air during each cycle.

After completing training, flies were relocated to a T-maze followed by 2 min of acclimation before behavioral testing. Flies loaded into the T-maze were descended to the decision chamber. Control and transgenic flies were given 2 min to choose between a light or dark chamber, after which the chambers were sealed to prevent further decision making and the proportion of flies in each chamber was recorded. Each assay was performed in at least 4 independent cohorts for each genotype and treatment group. Similarly, untrained cohorts of control and transgenic flies were loaded in the T-maze and were given 2 min to choose between dark and light chambers to confirm flies’ natural preference for light in the absence of training.

### 2.4. Climbing Assays

Climbing performance in control and AD flies was evaluated using a negative geotaxis assay. This assay takes advantage of adult flies’ natural tendency to migrate upward against gravity when agitated [25,29]. Young age-matched transgenic and control male flies treated or not with 1 mM lisinopril were included in each cohort. Two independent sets of experiments were performed. For each experiment, cohorts of at least 10 flies were transferred from standard medium into testing vials and allowed to acclimate for 2 min. After acclimation, flies were tapped to the bottom of the vial and given 10 s to pass an 8 cm mark. Each cohort repeated this climbing assay 5 times and the number of flies passing the 8 cm line in each trial was recorded. Trials were averaged and data were expressed as the proportion of all flies passing the 8 cm mark per genotype and treatment groups.

### 2.5. Measurement of Tryptophan Metabolites

Heads were dissected, immediately frozen in liquid nitrogen and stored at −80 °C until processing. Five independent replicates (30 heads/sample) were collected per genotype and treatment group. Heads were homogenized in 300 μL of ultrapure water containing 0.01% BHT (butylated hydroxytoluene; Sigma-Aldrich, Milwaukee, WI, USA). Metabolites in the control fly homogenate and calibration standards were prepared in ultrapure water containing 0.01% BHT (100 µL) with the appropriate amount of tryptophan (TRYP), kynurenic acid (KYNA), kynurenine (KYN) and 3-hydroxykynurenine (3-HK) to achieve concentrations ranging from 0.5 to 1000 ng/mL homogenate of each analyte. TRYP, KYNA, KYN, and 3-HK were purchased from Sigma-Aldrich (Milwaukee, WI, USA). Additional QCs were prepared in control homogenate to ensure accuracy of extraction from the sample matrix. Standards, QCs and samples were treated with deuterated internal standards (1 µg/mL TRYP-d5, KYNA-d5, KYN-d4, and 3-HK ^13^C_2_^15^N; Toronto Research Chemicals, Ontario, Canada) and 10 µL of 20% perchloric acid (Fisher Scientific, Waltham, MA, USA) to precipitate the proteins. After centrifugation for 5 min at 21000xg, the supernatant was transferred to an autosampler vials and analyzed in the positive ion mode by LC–MS/MS.

The LC–MS/MS system consisted of a Shimadzu system (Columbia, MD, USA) equipped with LC20-AD dual HLPC pumps, an SIL20-AC HT autosampler, and a DGU-20A2 in-line degasser. Detection was performed using an Applied BioSystems 4000 QTRAP (Applied Biosystems, Foster City, CA, USA) triple quadrupole mass spectrometer operated in the positive ion mode. Mass calibration, data acquisition and quantitation were performed using Applied Biosystem Analyst 1.6.2 software (Applied Biosystems, Foster City, CA, USA). Separation of the tryptophan metabolites and the internal standards from the homogenate matrix was achieved using a Phenomenex Luna Omega Polar C18, 100 × 2 mm 5 µm particle column (Phenomonex, Torrance, CA, USA). The mobile phase was delivered at a flow rate of 400 µL/min using a gradient elution profile consisting of DI water with 0.1% formic acid (Fisher Scientific, Waltham, MA, USA) (A) and acetonitrile (Fisher Scientific, Waltham, MA, USA) with 0.1% formic acid (B). A gradient elution profile was used in which mobile phase B was held at 3% for 2.5 min, then increased to 90% over 2.5 min, held at 90% for 1 min, returned to 3% and equilibrated for 5 min. The analytes were detected using multiple reaction monitoring (MRM) for the following the following transitions: TRYP (*m*/*z* 205.0→188.0), KYNA (*m*/*z* 190.1→144.0), KYN (*m*/*z* 209.2→192.0), 3-HK (*m*/*z* 225.0→110.0). The internal standard transitions are as follows: TRYP-d5 (*m*/*z* 210.0→192.0), KYNA-d5 (*m*/*z* 195.1→149.0), KYN-d4 (*m*/*z* 213.0→196.0), 3-HK ^13^C_2_^15^N (*m*/*z* 228.0→110.0).

### 2.6. Measurement of Thoracic H_2_O_2_ Levels

Thoracic H_2_O_2_ levels were measured as previously reported in [26]. Briefly, thirty thoraces per genotype and treatment groups were dissected between 10:00 a.m. and 11:00 a.m. from live control and transgenic flies treated or not with 1 mM lisinopril. The Fluorometric Hydrogen Peroxide Assay Kit (Sigma-Aldrich#MAK165-1KT, St. Louis, MO, USA) was used to quantify thoracic H_2_O_2_ levels as per the manufacturer’s instructions.

### 2.7. Statistical Analysis

Two-way analysis of variance (ANOVA) models were run to analyze the data, with genotype (AD or control), treatment (untrained or trained/trained plus lisinopril for cognitive behavior; no lisinopril or lisinopril for the other dependent variables), and genotype-by-treatment interaction terms included in the model. Block was used as a covariate in the model for climbing behavior to adjust for temporal differences in the two independent experiments. A two-way analysis of covariance (ANCOVA), with TRYP levels included as covariate in the models, was used to analyze variation for TRYP-derived metabolites. A log_10_ transformation was applied to the data that did not meet the assumption of normality. The Tukey test for post hoc pairwise comparisons was implemented to assess significant differences between groups. Statistical analyses were performed with SAS version 9.4 (SAS Institute, Cary, NC, USA). A significant level of 0.05 was used throughout this study.

## 3. Results

### 3.1. Lisinopril Improves Learning and Memory Impairment in AD Flies

**Figure 1:**
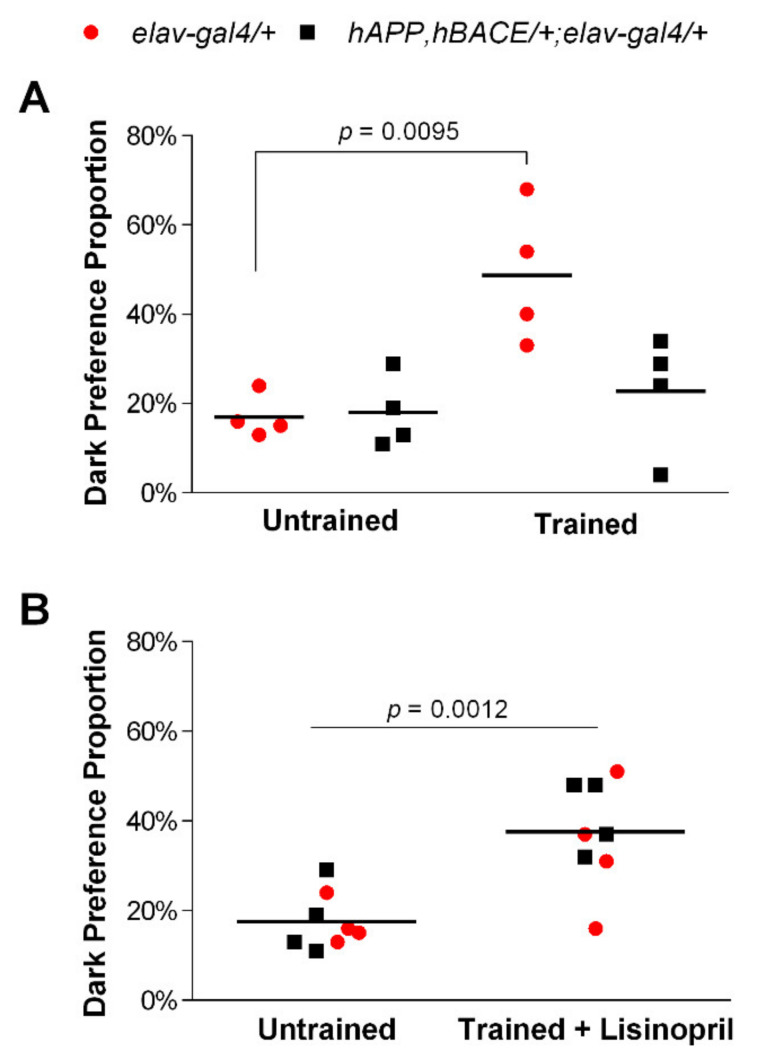
Lisinopril treatment mitigates learning and memory impairments in AD flies. (**A**) Control *elav-gal4*/*+* flies but not *hAPP*, *hBACE*/*+*; *elav-gal4*/*+* AD flies prefer moving significantly more towards a dark chamber than a light chamber after training. (**B**) A higher proportion of lisinopril-fed flies chose a dark chamber after training, independent of genotype. *p*-values obtained from Tukey–Kramer post hoc tests for multiple comparisons.

First, we tested whether untreated AD flies performed like the age-matched control flies in the aversive phototaxic suppression assay. Two-way ANOVA showed a significant effect of both genotype (F_1,12_ = 4.98; *p* = 0.0456) and training (F_1,12_ = 10.60; *p* = 0.0069) on preference towards dark. However, there was also a statistically significant genotype-by-training interaction effect (F_1,12_ = 5.80; *p* = 0.0330). As shown in Figure 1A, while no differences were found in *hAPP*, *hBACE*/*+*; *elav-gal4*/*+* AD flies, a significantly higher (65%) proportion of *elav-gal4*/*+* control flies moved towards the dark after training. Thus, AD flies displayed a learning and memory impairment.

Next, we assessed whether administration of lisinopril could improve the cognitive deficit in the AD flies. There was a statistically significant effect of training plus drug (F_1,12_ = 17.59; *p* = 0.0012) on preference towards dark, independent of genotype (F_1,12_ = 0.79; *p* = 0.3903). As reported in Figure 1B, both trained lisinopril-treated AD and control flies significantly increased their dark preference compared to their untrained counterparts. Thus, lisinopril treatment ameliorated learning and memory impairment in a *Drosophila* model of AD.

### 3.2. Lisinopril Improves Climbing Ability in AD Flies

**Figure 2:**
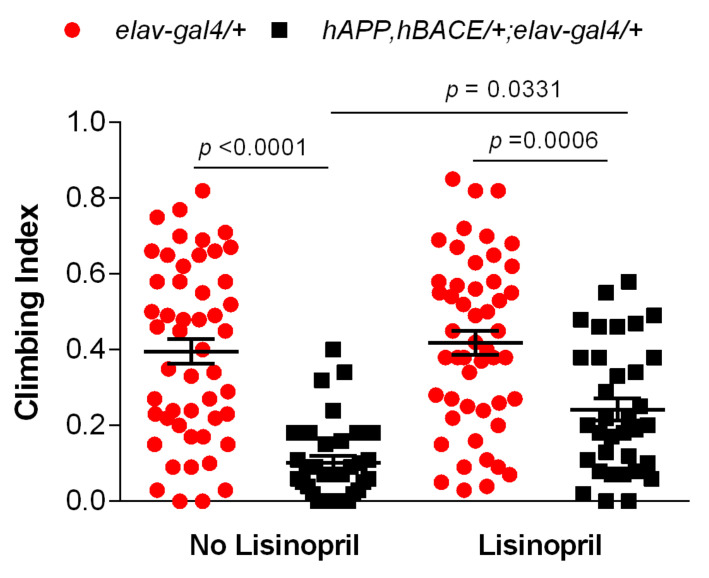
Lisinopril treatment improves climbing ability in AD flies. On average, untreated *hAPP*, *hBACE*/*+*; *elav-gal4*/*+* AD flies traveled significantly less than matched controls. However, while climbing is not affected by the ACEi treatment in controls, lisinopril-fed AD flies traveled significantly more than untreated AD flies. Error bars represent the S.E.M. *p*-values obtained from Tukey–Kramer post hoc tests for multiple comparisons.

Previous work showed that the *hAPP*, *hBACE*/*+*; *elav-gal4*/*+* AD flies used in this study display motor reflex behavior abnormalities [25]. As such, we sought to test whether lisinopril administration might improve their climbing ability. Two-way ANOVA showed significant effects of genotype (F_1,163_ = 63.47; *p* < 0.0001) and treatment (F_1,163_ = 6.92; *p* = 0.0093) on climbing behavior, but also a significant interaction between genotype and treatment (F_1,163_ = 4.09; *p* = 0.0449), indicating that lisinopril treatment affected climbing ability in a genotype-specific manner. As shown in Figure 2, both untreated and treated controls performed significantly better than untreated AD flies, but there was no difference in climbing ability between untreated and treated controls. On the other hand, lisinopril-treated AD flies experienced a 2-fold improvement in climbing ability compared to their untreated counterparts. Thus, lisinopril treatment showed beneficial effects on physical movement in a *Drosophila* model of AD.

### 3.3. Lisinopril Lowers Thoracic H_2_O_2_ Abundance in AD Flies

**Figure 3:**
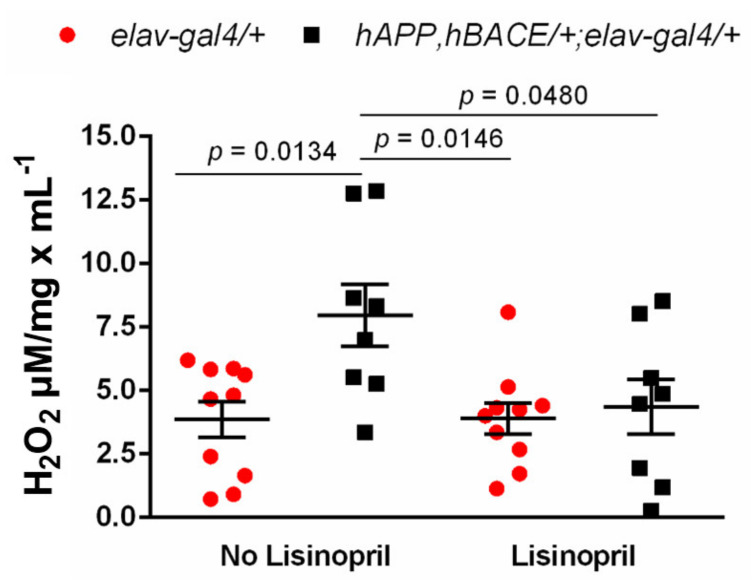
Lisinopril reduces the abundance of reactive oxygen species in muscle-enriched thoraces of AD males. Untreated *hAPP*, *hBACE/+*; *elav-gal4/+* AD flies displayed higher thoracic H_2_O_2_ levels than untreated and lisinopril-fed *elav-gal4/+* controls. Lisinopril treatment significantly decreased the levels of H_2_O_2_ in AD flies compared to the untreated AD flies. Error bars represent the S.E.M. p-values obtained from Tukey–Kramer post hoc tests for multiple comparisons.

Growing evidence suggests that the benefits of RAS blockade with ACEis can be attributed to its ability to decrease oxidative stress [30], a hallmark in normal aging [31] and AD pathophysiology [32]. Previously, we reported that lisinopril treatment reduces thoracic H_2_O_2_ levels [26]. Given that thoraces are mainly enriched in skeletal muscles, we reasoned that lisinopril might improve climbing ability in AD flies by reducing muscle H_2_O_2_ levels. Two-way ANOVA revealed a significant effect of genotype (F_1,32_ = 6.51; *p* = 0.0157) on thoracic H_2_O_2_ levels, with AD flies having overall higher levels of H_2_O_2_ than controls. There was no statistically significant effect of treatment (F_1,32_ = 4.04; *p* = 0.0530), but the analysis showed a significant genotype-by-treatment interaction effect (F_1,32_ = 4.23; *p* = 0.0479), indicating that the effect of lisinopril on thoracic H_2_O_2_ levels was genotype-specific. As shown in Figure 3, while no differences were observed between thoracic H_2_O_2_ levels of untreated and lisinopril-treated *elav-gal4*/*+* control flies, untreated *hAPP*, *hBACE*/*+*; *elav-gal4*/*+* AD flies displayed a ~2-fold increase in H_2_O_2_ levels compared to untreated controls. However, lisinopril-treated AD flies had significantly lower (45%) levels of H_2_O_2_ than the untreated AD flies. Thus, our results suggest that the positive impact of lisinopril on physical function might be, in part, explained by a significant reduction in ROS levels in the thoraces of the lisinopril-fed AD flies.

### 3.4. AD Flies Have Significantly Higher Levels of 3-HK in Their Heads

In humans, the KP of tryptophan metabolism has been reported to be differentially activated in neuroinflammatory diseases, including AD [33]. Previous work also showed that ACEi, including lisinopril, influence KYNA production in rat brain cortex in vitro [34]. Based on these observations, we next assessed whether the KP was altered in our *Drosophila* AD model and whether lisinopril treatment had any effect on the production of KP metabolites. Table 1 reports the results of the ANCOVA analyses used to assess variation for TRYP-derived metabolites. There was no effect of either genotype or treatment on head levels of TRYP, KYNA and KYN levels. However, there was a statistically significant effect of genotype on the levels of 3-HK.

**Figure 4:**
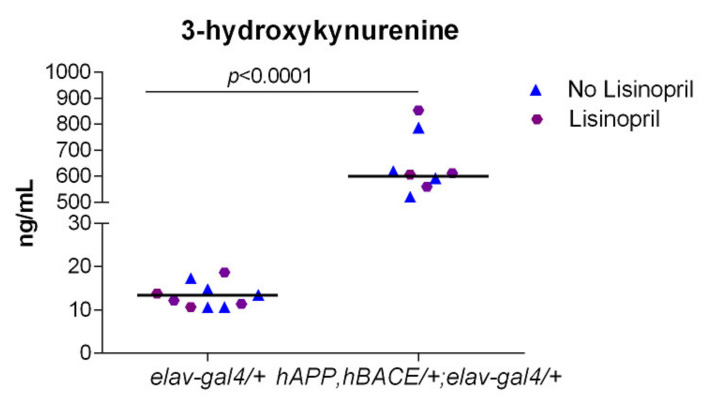
The kynurenine pathway metabolite 3-hydroxykinurenin (3-HK) is highly produced in AD flies. Concentrations of the neurotoxic 3-HK in the heads of *hAPP*, *hBACE*/*+*; *elav-gal4*/*+* AD flies were dramatically higher than in control flies, independently of whether flies were fed lisinopril on not. *p*-value obtained from Tukey–Kramer post hoc tests for multiple comparisons. Tryptophan levels were used as a covariate in the analysis. Data points show unadjusted values.

Regardless of treatment, *hAPP*, *hBACE/+*; *elav-gal4/+* AD flies displayed ~33-fold higher levels of 3-HK in their heads than *elav-gal4/+* flies (Figure 4). Thus, our results suggest that AD flies produce high levels of the neurotoxic 3-HK but lisinopril does not influence metabolite production.

## 4. Discussion

Little progress has been made in identifying effective therapies for progressive memory loss, a hallmark symptom in AD [35]. Like other tissues, the brain has its own RAS system with the ability to synthesize all the main RAS biologically active peptides, including Ang II [30]. Prior clinical studies have shown that the use of centrally acting ACEis, such as captopril, perindopril, and lisinopril, can improve cognitive function outcomes and slow down the development of AD hypertensive patients [13,31]. These effects appear to be independent of the drug’s blood pressure-lowering properties. Yet, the mechanisms behind the beneficial effects of the drugs remain unknown, mostly because disentangling their vascular hemodynamic effects from their direct effects on local RAS components remains a challenge in humans and in vivo vertebrate models [32]. As in humans, in this study, we showed that the ACEi lisinopril ameliorated the learning and memory deficits of AD flies.

Recently, a 28 day treatment with the ACEi captopril, another central-acting ACEi, proved to be successful in improving short-term memory in a moderately compromised fly model of AD. However, the study failed to do so in the more severely compromised Aβ42 fly model [23]. The latter is comparable to the model we used in our study, with neuropathologies and memory defects established within days, as described previously [25]. This accounts for the striking memory and physical function deficits that were evident as early as 5–7 days post-eclosion in our AD cohorts. Whether lisinopril intervention is successful at ameliorating memory deficits in older AD flies remain to be determined. Additional evidence to support the link between lisinopril and cognitive function in other models of AD comes from studies in a non-transgenic streptozocin-induced rodent model of AD which demonstrated that lisinopril intervention reduced learning and memory deficits in AD animals [36]. However, the validity of this model for preclinical testing of therapeutic candidates for AD has been recently challenged [37]. Our results provide strong evidence that unlike captopril, lisinopril had the ability to postpone memory deficits in a more severely compromised fly model of AD, a well-described powerful in vivo resource for the screening of novel therapeutic candidates in AD [38].

Besides the positive impact on learning and memory, we found that lisinopril postponed the decline in physical performance in our *Drosophila* model of AD. Our results echo findings from Gabrawy et al. [22], who reported that lisinopril decelerated age-dependent decline in climbing speed, endurance, and strength in *D. melanogaster*. Furthermore, they align with various clinical studies associating ACEi administration to an improved exercise capacity and muscle strength [39,40,41]. However, genetic variation has also been reported to play a role in the response to ACEis among hypertensive patients [42]. Similarly, it has been shown that the effect of lisinopril treatment on measures of physical performance is differentially affected by genotype and age among naturally-derived inbred lines of *Drosophila melanogaster* [22]. Such similarities highlight the evolutionary conservation of ACE across species making *D. melanogaster* a powerful model to study the relationship between ACEi therapy and the progression of physical deficits in the context of AD.

An additional finding of our study is that the benefits of lisinopril on climbing ability in AD flies are likely mediated by a lisinopril-induced reduction in thoracic H_2_O_2_. This is not surprising since oxidative stress is one of the most common mechanisms of muscular mass decline [43], which leads to defects in physical performance. Our findings also support our recent work showing that lisinopril reduced *Drosophila* thoracic H_2_O_2_ levels and positively impacted various mitochondrial bioenergetic traits in wild-type *Drosophila* lines [26]. Experimental evidence in animals suggests that Ang II supplementation increases oxidative stress production in skeletal muscle with a deleterious impact on exercise capacity [44]. While several lines of evidence support the antioxidant capacity of ACEis in various tissues [45,46], the evidence in skeletal muscle mostly restricts to models of muscular dystrophy [47]. ACEis counteract the Ang II-mediated dysregulation of a variety of physiological pathways to influence physical function but mechanisms are not well understood. Thus, our results add to a growing body of evidence suggesting that the benefits of ACEis on physical performance may extend to other models of age-associated muscular degeneration, such as AD, and may be mediated by lisinopril-induced decrease in ROS production. Of note, the benefits of lisinopril on motor function were genotype-specific and limited to young AD flies. This is not unexpected since functional changes in fly skeletal muscle have been shown to manifest early in age-related pathologies such as AD [48].

Emerging research suggests that the KP of tryptophan metabolism, a key regulator of neuroinflammation, is differentially activated in AD [49,50,51]. The KP is the main route of tryptophan catabolism and it generates the metabolite 3-HK, which has been associated to the generation of oxidative stress and neuronal death [52,53]. Moreover, levels of the neurotoxic 3-HK have been reported to be strikingly increased in the serum of AD patients [51]. On the other hand, KYNA opposes the excitotoxicity-mediated actions on neuronal cells [54] and has a neuroprotective role [55,56], with lower levels found in patients with AD [57]. As in humans, this pathway encompasses key signaling molecules with neuroregulatory effects in fly brains [58]. Enhancing the production of 3-HK [59] or increasing the levels of KYNA [58] has been associated to neurodegeneration and neuroprotection, respectively, in fly models of neurodegeneration. Although we did not find any effect of lisinopril on KP metabolites, our study is the first, to our knoweledge, to demonstrate that young AD flies had strikingly higher levels of 3-HK in heads compared to controls. Brain perfusion studies in rodents suggest that such high levels in heads may derive from high circulating peripheral levels [60], as described in patients with AD [51]. Indeed, the kynurenine-derived 3-HK has been highlighted as a potential early stage biomarker in AD [51]. Recent evidence suggests that the cytotoxic effects of 3-HK may represent the mechanistic linkage between mild cognitive impairment with age-associated chronic inflammation and frailty [20]. Further, increased levels of 3-HK have been reported to induce impairments of middle-term memory in *D. melanogaster* [61]. It is, therefore, plausible that the high levels of 3-HK in the AD flies might be involved in their impairment in cognitive and physical functioning. Additional studies are, however, needed to validate this hypothesis.

Recent studies highlight the role of Ang1–7 and AngIV [62,63] as RAS components with potential neuroprotective roles in AD. Lisinopril has been shown to augment plasma levels and urinary excretion rates of Ang(1–7) in rodent models [64]. Likewise, it is argued that therapeutic approaches targeting AngII, such as lisinopril, may alter the synthesis of AngIV [65]. Whether any of the beneficial effects of lisinopril involve changes in the levels of Ang (1–7) or AngIV is yet to be determined.

## 5. Conclusions

In conclusion, our findings provide compelling evidence of the therapeutic potential of the ACEi lisinopril in the treatment of the decline in cognitive and physical functioning occurring in a fly model of AD. Benefits of lisinopril on physical performance may derive from a lisinopril-mediated decrease in the levels of thoracic H_2_O_2_. Furthermore, although lisinopril did not affect the levels of the neurotoxic 3-HK metabolite, the finding of its high production in our *Drosophila* AD model is in line with previous work in humans and strengthens its value as a promising early disease biomarker. Overall, our results confirm that *Drosophila* is a powerful model to elucidate the underlying mechanisms mediating the beneficial effect of lisinopril on the symptoms of AD. Determining whether lisinopril confers similar benefits in females and/or later in life is beyond the scope of this manuscript and will be the focus of future investigation.

## Figures and Tables

**Table 1:** Analysis of Covariance for kynurenine pathway metabolites in the heads of AD and control flies.

Metabolite	Source ^a^	Df	MS	F-Value	*p*-Value
TRYP ^†^	Genotype	1	0.013	0.64	0.4356
	Treatment	1	0.014	0.66	0.4269
	Genotype × Treatment	1	0.011	0.54	0.4728
	Error	16	0.020		
KYN	TRYP	1	134.311	13.05	0.0026
	Genotype	1	15.567	1.51	0.2377
	Treatment	1	3.374	0.33	0.5754
	Genotype × Treatment	1	0.740	0.07	0.7922
	Error	15	10.291		
KYNA^†^	TRYP	1	0.040	0.59	0.4547
	Genotype	1	0.017	0.25	0.6217
	Treatment	1	0.022	0.33	0.5761
	Genotype × Treatment	1	0.140	2.07	0.1706
	Error	15			
3-HK	TRYP	1	62,309.255	8.69	0.0100
	Genotype	1	1,549,853.363	216.27	<0.0001
	Treatment	1	815.481	0.11	0.7405
	Genotype × Treatment	1	888.440	0.12	0.7297
	Error	15	7166.188		

^a^ Source of variation. Df: degrees of freedom. MS: mean squares. ^†^ Data were log_10_ transformed to meet the assumption of normality. TRYP: tryptophan; KYN: kynurenine; KYNA: kynurenic acid; 3-HK: 3-hydroxykynurenine. TRYP levels were used as a covariate in the analysis.

## Data Availability

All the data provided in the manuscript and Supplementary Materials.

## Supplementary Materials

The following is available online at https://www.mdpi.com/article/10.3390/pathophysiology28020020/s1, Figure S1: Lisinopril concentration in whole-body homogenates.
