# Supplementary material for: The Angiotensin-Converting Enzyme Inhibitor Lisinopril Mitigates Memory and Motor Deficits in a Drosophila Model of Alzheimer’s Disease
Source: Pathophysiology. 2021 Jun 18;28(2):307–19. doi: 10.3390/pathophysiology28020020 (PMC8830455; doi:10.3390/pathophysiology28020020)
Supplement: Supplementary file 1 [file pathophysiology-28-00020-s001.zip › pathophysiology-1160740-supplementary.pdf]

Supplemental Figure

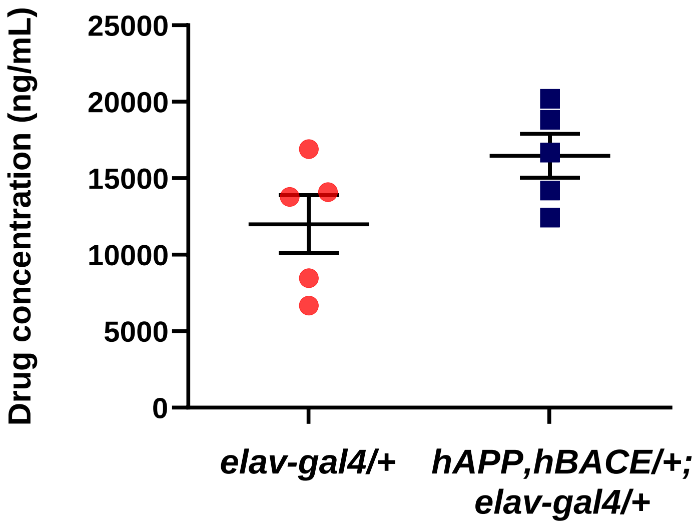

Figure S1. ***Lisinopril concentration in whole body homogenates.*** Concentration of lisinopril was not different between genotypes. Data points show unadjusted values.
